# Low-cost automatic temperature monitoring system with alerts for laboratory rearing units

**DOI:** 10.1016/j.mex.2019.09.013

**Published:** 2019-09-13

**Authors:** François Rebaudo, Romain Benoist

**Affiliations:** UMR 247-9191 EGCE, IRD, CNRS, Univ Paris Saclay, Gif-sur-Yvette, France

**Keywords:** Automatic system for monitoring temperature in laboratory insect rearing units, Raspberry Pi, Temperature, Sensor, Insects

## Abstract

Monitoring accurately temperature is a key issue in biological studies involving living experimental material. It is especially true for insects which body temperature is mostly controlled by environmental temperature, with profound consequences of a few degrees variation on most physiological processes such as survival, development, fecundity, and mobility. If programmable rearing units can be purchased, it remains important to monitor and store temperature information acquired inside the rearing unit to ensure that observed phenomena are not the result of unintended and not scarily noticeable changes in temperature, and to account for the effect of temperature variation in statistical analysis. As most laboratories involved in insect rearing dispose of a large number of rearing units, the technical solution should meet the monitoring needs while being affordable and adaptable to various experimental designs. For that purpose, we designed a low cost (below 100€) and open source automatic temperature monitoring system for rearing units in laboratory. Key features providing advantage over pre-existing methods include:

• Highly configurable temperature monitoring and life-time storage capacity
• Email alerts based on configurable user-defined threshold
• Automatic configurable reports in the form of dashboards

## Graphical abstract

**Figure:**
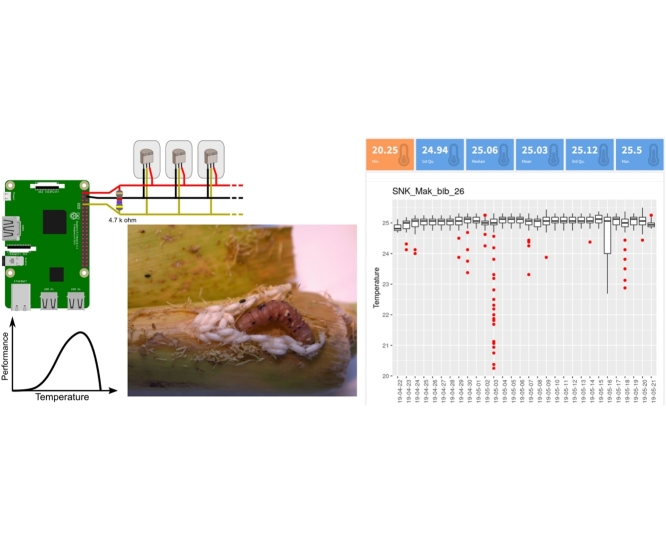

**Specification Table**

**Table:** 

Subject Area:	Agricultural and Biological Sciences
More specific subject area:	Insect science (experiments and rearing units in laboratory)
Method name:	Automatic system for monitoring temperature in laboratory insect rearing units
Name and reference of original method:	Lewis AJ, Campbell M, Stavroulakis P (2016) Performance evaluation of a cheap, open source, digital environmental monitor based on the Raspberry Pi. Measurement 87:228–235. doi: 10.1016/j.measurement.2016.03.023
Resource availability:	Hardware: Raspberry Pi 3 model B+ MicroSD card 8 Go (16 Go recommended) DS18B20 temperature sensors Electric cables, tin and soldering iron Software: Linux, Python, R

## Method details

### Monitoring and recording temperature in rearing units

When designing a scientific experiment under controlled conditions, it is not only necessary to ensure that environmental conditions are actually controlled, but it is also needed to record these environmental conditions so that potential environmental conditions variation can be included in the analysis. In insects, temperature is the key driver of most physiological processes such as temperature-dependent development, which drives the timing of the insect phenological stages [1]. Furthermore, some insect species are very sensitive to variations in temperature, or to fluctuating temperature regimes [3]. Hence, there are many examples on why it is important to monitor and record temperature for experiments in rearing units with insects. If insects of the same population are kept in two different rearing units at the same constant temperature, it is required to be able to separate the effect of intraspecific variability of development in response to temperature [2], from the effect of unintended variations in temperature. If populations are to collapse it is required to quickly identify potential causes and put aside environmentally controlled factors such as temperature. Such monitoring can also be of importance in evaluating the impact of insect manipulation on temperature when opening and closing the rearing unit doors, or to test for the homogeneity of temperature inside the rearing unit. In mentioned examples, the temperature information needs i) to be monitored inside the temperature rearing unit and easily positioned at different places; ii) to be recorded at configurable time steps and stored over the duration of the experiment and beyond.

In case of failure of a rearing unit, it is convenient to dispose of the complete historical data on temperature inside the rearing unit to and an alarm is also needed. Indeed, it can be required to act quickly so as to maintain the biological material, especially in the case of the conservation of an endangered species or a strain. Furthermore, the system should be independent from the rearing unit electrical circuit to avoid a general blackout if the failure is associated with an electric malfunction.

In this article we present the automatic temperature monitoring system that we developed for rearing unit, based on a published temperature monitoring system [4]. This system provides an alarm system and a dashboard html report that allow visualizing the temperature inside rearing units.

### Building and deploying the temperature monitoring system

#### Hardware

The hardware used to collect and analyze the temperature information is a Raspberry Pi 3 model B + with a 16 Go micro SD card. The Raspberry Pi is a low-cost computer of the size of a credit card (approximately 40€, SD card included). The main advantage is that it operates using Linux, which is a common operating system to the scientific community. The official supported operating system (OS) is Raspbian which can be install in a micro SD card from the Raspberry Pi foundation (https://www.raspberrypi.org/; using another computer than the Raspberry Pi). Once the OS is installed on micro SD card, the Raspberry Pi can be started. The system should be updated from the terminal using:

sudo apt-get update

sudo apt-get upgrade

In the configuration menu (sudo raspi-config), from "Interfaces", SSH and 1-wire should be enabled. In "Localization", the time zone and Wi-Fi country (if required) should be updated to correspond to the computer localization. It is also advised to change the default password and reboot the computer so that changes can take effect. Setting a fix IP address is also advised to easily communicate with the computer from SSH protocol (an SSH connection allows to control the Raspberry Pi from any other computer so that it is no longer necessary to have a screen, mouse and keyboard plugged in to the Raspberry Pi). Depending on the network configuration, setting a fix IP address may require the intervention of the IT services. For convenience it is assumed in this article that the fix IP address of the Raspberry Pi is “192.168.0.30”. The Raspberry Pi need to be connected to Internet either through the Ethernet port or through Wi-Fi.

For the temperature monitoring, DS18B20 sensors (Maxim Integrated Products, Inc) are used. DS18B20 sensors are digital thermometers (slaves) that communicate with a central microprocessor (master: the Raspberry Pi) over a 1-Wire bus. Each DS18B20 has a unique 64-bit serial code which is practical to connect multiple sensors on the same 1-Wire bus. Temperature measurements range from -55 °C to +125 °C with a ±0.5 °C accuracy over a temperature range from −10 °C to +85 °C. A complete description can be found on the manufacturer web site (https://www.maximintegrated.com).

**Fig. 1:**
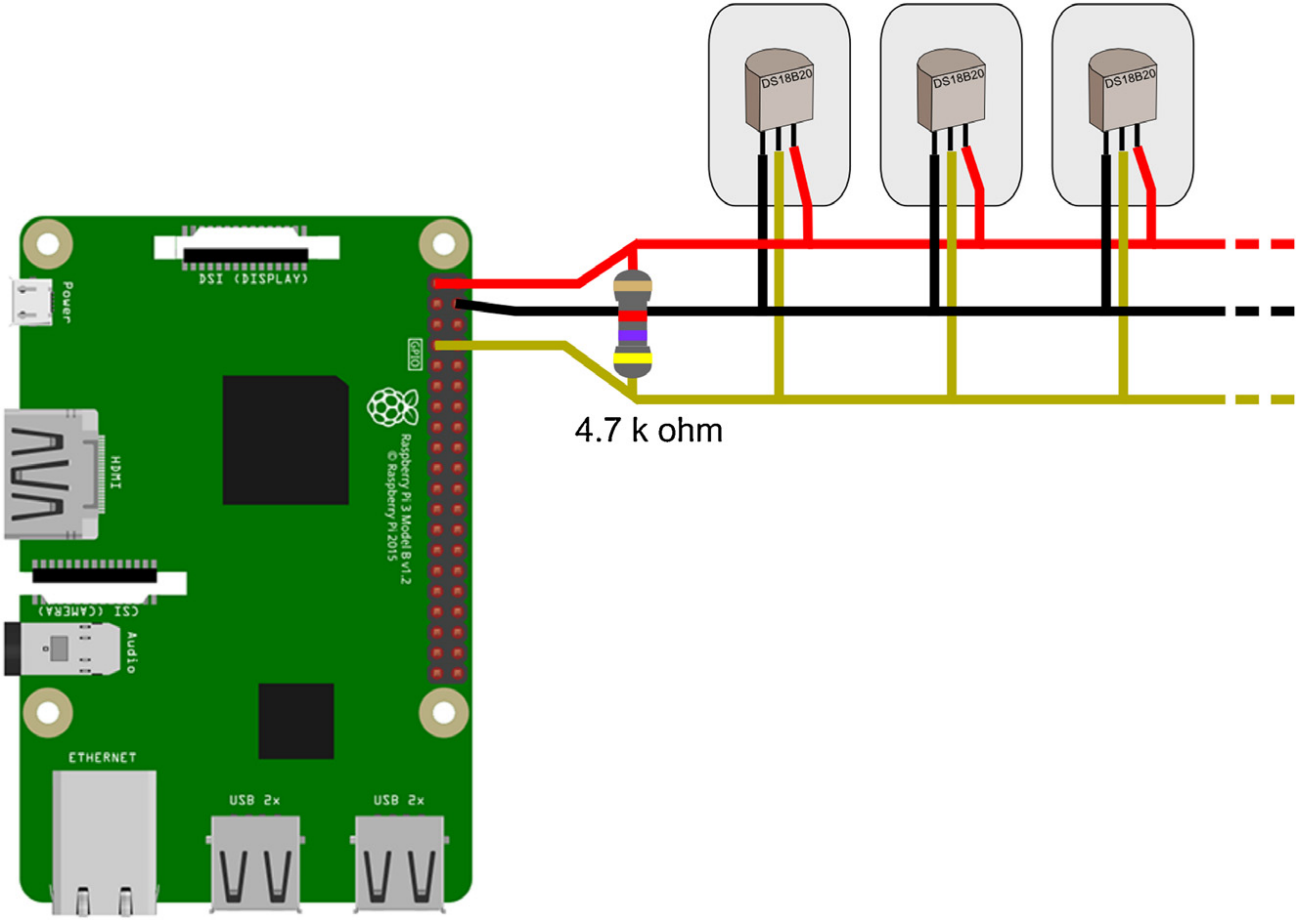
Schematic representation of the circuit. The Raspberry Pi is connected to Internet using the Ethernet port (note that Raspberry Pi 3 model B + has a Wi-Fi card).

The sensors can be connected in parallel to the Raspberry Pi using the GPIO4, Ground, and 3V3 power pins, with a 4.7 k ohm resistor between the GPIO4 and the 3V3 power (see Fig. 1). DS18B20 sensors can be connected using jumper wires, but it is advised to solder the sensors to wires for long term usage.

#### Software

**Fig. 2:**
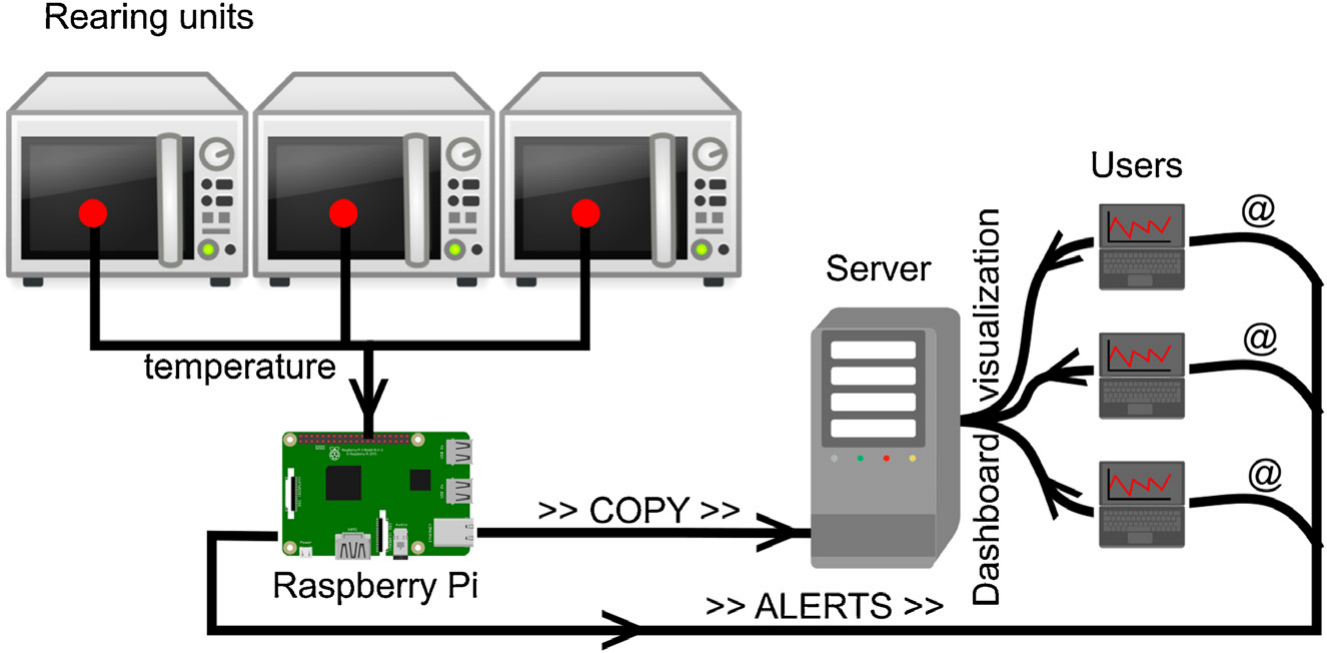
Schematic representation of the monitoring system.

An overall schematic representation of the monitoring system can be found in Fig. 2.

##### Reading temperature from the DS18B20 sensors and store information into CSV files

All the procedures to read the temperature and store the information into a CSV file uses Python 3.5.3. The complete script can be found in Supplementary material S1 ("envColTemp_XX.py"). A CSV file is created with the headers corresponding to the data to be stored ("year", "month", "day", "hour", "minute", "second", "sensorId", "temperature"; with sensorId corresponding to the DS18B20 64-bit serial code), and named after the date of initialization.

Temperature reading is contained in the "/sys/bus/w1/devices/" directory of the Raspberry Pi, under directories starting with "28-00". The Python script used to retrieve the temperature information was obtained and adapted from a StackOverflow post by "joan" under a CC-BY-SA license (https://raspberrypi.stackexchange.com/).

**Figure:**
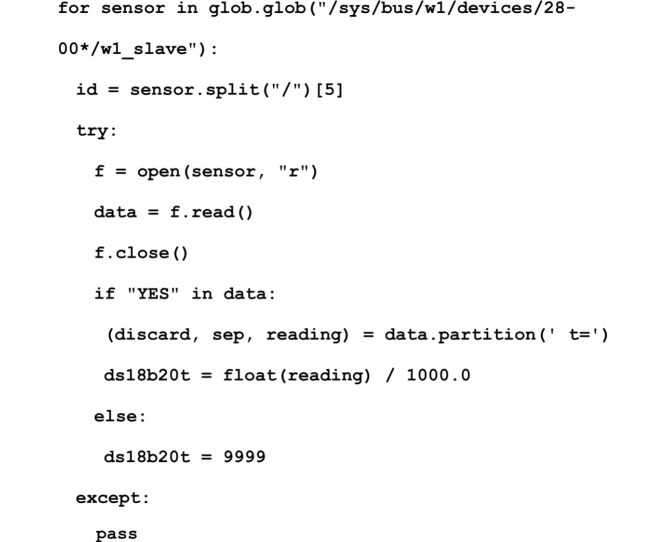

In this article temperature is measured every minute using "time.localtime() [5] = = 0″ (line 54 in Supplementary material S1). To reduce the number of writing operations on the micro SD card, the temperature information is stored in the RAM with a default number of reading of 40 that can be changed using "RAMReadNumber" variable (line 21). After 40 reads, the data is transferred to the csv file. To reduce the management of large files by the Raspberry Pi, a new file is created once the current file exceeds 10 Mo, configurable with the variable "fileMaxSizeMo" (line 22).

Once uploaded into the Raspberry Pi (here in the "Documents" directory), from the command line interface of the Raspberry Pi (e.g., through an SSH connection), a bash file can be created to launch automatically the script at each computer startup using a CRON job. This is particularly important in case of power failure or when installing the monitoring system: the monitoring will start automatically and power failure can also be monitored. A separate bash script is used to launch the monitoring system automatically (Supplementary material S2, "launcher.sh"), as it allows to maintain different versions of the Python script without having to change the CRON job.

nano launcher.sh # create and edit bash file

chmod 755 launcher.sh

A directory "logs" was created under the "Documents" directory for the logs files. CRON job was created using "sudo crontab -e":

@reboot sh /home/pi/Documents/launcher.sh >/home/pi/Documents/logs/launcher_log 2 > &1

##### Configuring an alert system with emailing

A simple alert system triggered by a temperature over or below a threshold cannot be used in the case of a rearing unit because variations of temperatures that would trigger the alert are expected when opening the rearing unit for insect manipulation. Thus a more configurable threshold has been developed in which the alert is triggered in case the average temperature over a period of time reach the threshold. A different minimum and maximum threshold can be used for each sensor, like in the case presented here.

A Python script was used and uploaded into the directory "Documents" to be able to send emails automatically with a content defined by arguments passing through the script execution. The Python script can be found in Supplementary material S3 ("emailSender_XX.py").

Then, an R script [5] was made and uploaded into the directory "Documents" to configure the threshold (Supplementary material S4, " envCheckTemp_XX.R"). In this article the monitoring system had 8 DS18B20 sensors connected to the Raspberry Pi. Maximum temperature thresholds for the 8 sensors are stored in the variable "paramMaxTemp" and minimum thresholds in the variable "paramMinTemp". The average temperature for triggering alerts was set to 60 min with the variable "paramDuration". All CSV files are merged into a data frame R object, and sensors renamed from the manufacturer 64-bit serial code to a more human readable name (each sensor can be identifiable by sequentially holding them while recording temperature). If the average temperature over the defined period of time reach a threshold, then the python script is launched from R with arguments corresponding to the average temperature and the name of the sensor. In order to use the script, R must be installed in the Raspberry Pi:

sudo apt-get install r-base

In order to monitor automatically the temperature and check for potential anomalies, a bash script was used and launched automatically every hour using a CRON job (Supplementary material S5, "checker.sh").

nano checker.sh # create and edit bash file

chmod 755 checker.sh

CRON job was created using "sudo crontab -e":

* * * * sh /home/pi/Documents/checker.sh >/home/pi/Documents/logs/checker_log 2 > &1

The CRON job first five fields correspond to date and time, followed by the command. The date and time fields correspond to minute (0–59), hour (0–23), day of the month (0–31), month (0–12), and day of the week (0–7), respectively. The star symbol "*" represents all available values. Here "0 * * * * *" means that the command will be executed every time the minutes reach 0 (i.e., every hour). The complete documentation on CRON jobs can be found from any Linux terminal using "man crontab".

##### Additional security measures (optional)

A file backup system was used to ensure CSV files security. The backup is triggered from a server and not from the Raspberry Pi using a CRON job every day at 7.00 a.m.:

7 * * * python3 /xxx/copyToCommun.py >/xxx/copyFromCommun_log 2 > &1

The backup system consists in a Python script (Supplementary material S6, "copyToCommun.py") which lists the files in the Raspberry Pi and locally in the server. If new files or files with a bigger size are found in the Raspberry Pi, they are automatically copied to the server. It can also be triggered manually from the server at any moment. Using a server to store copies of the Raspberry Pi is useful to ensure long term storage, but also in the case of analyses that require a large amount of RAM or other computer resources not available from the Raspberry Pi.

##### Dashboard (optional)

**Fig. 3:**
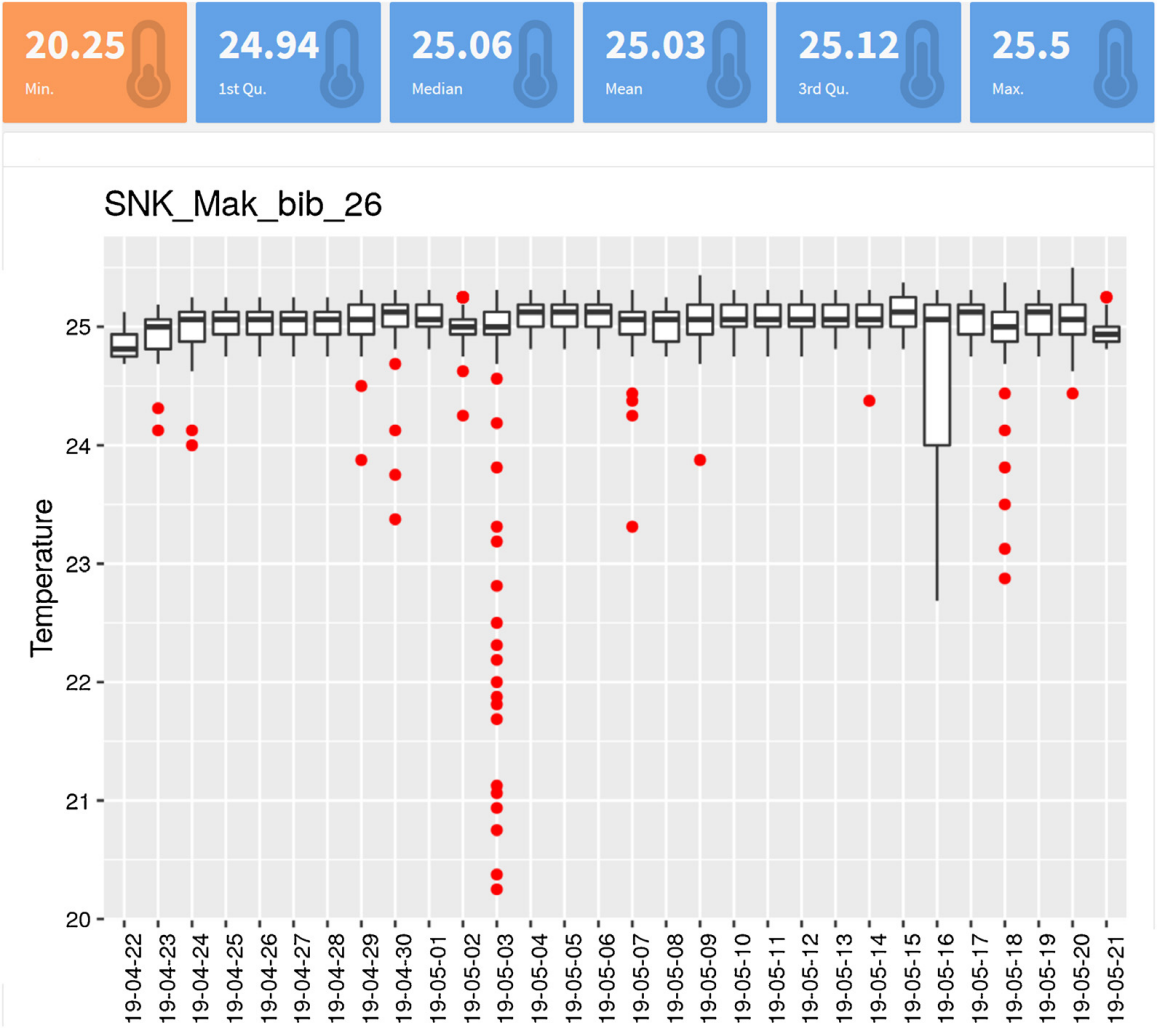
Example of a simple dashboard for one rearing unit (named "*SNK_Mak_bib_*26″ and set to 25°C) to visualize temperature within the rearing units for the last month. Daily temperatures (yy-mm-dd) are represented as boxplots and red dots represent outliers (they correspond to the times when the door of the rearing unit was opened; the door was opened multiples times on May 3rd and for a long time on May 16th). The dashboard is in HTML format so that it can be accessed from any Internet navigator by any operator with no prior experience in programming, and hosted in a local or distant server.

If the information about the temperature monitoring needs to be revised by several people with graphical visualizations, working with copies of the original files on a server rather than the Raspberry Pi itself is advised (see section 2.3.). In the case presented here, a dashboard in html is created every day at 7.30 a.m. in a server using R [5], with the packages "flexdashboard" [6] and "ggplot2″ [7] (Fig. 3). The complete script to build the dashboard and to compile it into html can be found in Supplementary material S7 and S8 ("dashboard_MONTH.Rmd", "renderRMDtoHTML.R").

7 * * * Rscript /xxx/renderRMDtoHTML.R >/xxx/dashboard_log 2 > &1

### Conclusion

This method article presents a simple yet powerful system for temperature monitoring at low cost and highly configurable particularly suitable for experiments in laboratory with insect rearing units. It is based on a Raspberry Pi computer and DS18B20 temperature sensors. With eight temperature sensors, the Raspberry Pi, the micro SD card, the power supply, and the wires, the overall cost is below 100€ which makes the system affordable and easy to deploy even in laboratories with a large number of rearing units. Automatic reports and email alerts can easily be configured to ensure full control over your experiments together with long term storage of temperature data, and easy access to the information without programming skills. All the files can be found in GitHub (https://github.com/frareb/raspi_tempMonitor).
