# Supplementary material for: Low-cost automatic temperature monitoring system with alerts for laboratory rearing units
Source: MethodsX. 2019 Sep 13;6:2127–33. doi: 10.1016/j.mex.2019.09.013 (PMC6812373; doi:10.1016/j.mex.2019.09.013)
Supplement: Supplementary file 1 [file mmc1.docx]

**Supplementary material**

All the files can be found in GitHub (https://github.com/frareb/raspi_tempMonitor).

**Supplementary material S1** (Python 3 script "envColTemp_XX.py"). Python script located in the Raspberry Pi to read the temperature information from the DS18B20 sensors and store it into CSV files.

**Supplementary material S2** (Bash script "launcher.sh"). A Bash script to run the Python "envColTemp_XX.py" script.

**Supplementary material S3** ("emailSender_XX.py"). A Python script to send emails automatically with content defined by arguments given to the script.

**Supplementary material S4** ("envCheckTemp_XX.R"). An R script to define thresholds triggering email alerts.

**Supplementary material S5** ("checker.sh"). A Bash script to run the R "envCheckTemp_XX.R" script.

**Supplementary material S6** ("copyToCommun.py "). A python script to backup Raspberry Pi files on a server.

**Supplementary material S7** ("dashboard_MONTH.Rmd "). An R script using markdown to produce a dashboard.

**Supplementary material S8** ("renderRMDtoHTML.R "). An R script to compute a HTML file from the R markdown file.
